# Translation, Cultural Adaptation, and Psychometric Validation of the Nursing Evidence-Based Practice Survey and the Evidence-Based Practice Self-Efficacy Scale in the Greek Language

**DOI:** 10.7759/cureus.85835

**Published:** 2025-06-12

**Authors:** Evangelos C Fradelos, Stella Zetta, Maria-Eleni Vasileiou, Vissarion I Bakalis, Pavlos Sarafis, Afroditi Zartaloudi, Maria Saridi, Maria Chatzi, Aikaterini Toska

**Affiliations:** 1 Laboratory of Clinical Nursing, Department of Nursing, University of Thessaly, Larissa, GRC; 2 Department of Primary Health Care, University of Thessaly, Larissa, GRC; 3 Department of Nursing, University of Thessaly, Larissa, GRC; 4 Department of Nursing, University of Thessaly, Lamia, GRC; 5 Department of Nursing, University of West Attica, Athens, GRC; 6 Department of Nursing, University of Thessaly, Larrisa, GRC; 7 Infectious Diseases Unit, University Hospital of Larissa, Larissa, GRC; 8 General Department of Lamia, University of Thessaly, Lamia, GRC

**Keywords:** clinical nursing, community nursing, evidence-based clinical practice, evidence-based practice, nursing practice

## Abstract

Background and aim: Evidence-based practice (EBP) improves the quality of healthcare services and enhances patient safety. However, in Greece, the adoption of EBP remains modest, while validated assessment tools in the Greek language for measuring EBP among nurses are limited. The purpose of the study was to translate, culturally adapt, and psychometrically validate the Nursing Evidence-Based Practice Survey and Evidence-Based Practice Self-Efficacy Scale (EBP SES) tools into Greek.

Methodology: The study sample comprised 222 registered nurses, including both clinical nurses and school-based nurses, through convenience sampling. Reliability analyses (Cronbach’s α) and confirmatory factor analysis (CFA) were conducted to investigate internal consistency and structural validity.

Results: The instruments showed high reliability (Cronbach’s α = 0.78-0.96). The mean value of the Nursing Evidence-Based Practice Survey was 3.88 (standard deviation [SD] = 0.66), and the EBP SES was 73.4 (SD = 14.0), which reflects an overall favorable perception of EBP engagement. In CFA, the five-factor model of the Nursing EBP Survey showed fit indices: Goodness of Fit Index (GFI) = 0.960, Standardized Root Mean Square Residual (SRMR) = 0.041, Comparative Fit Index (CFI) = 0.899, Tucker-Lewis Index (TLI) = 0.885, and χ²/df = 4.1, while the single-factor model of the SES showed GFI = 0.980, SRMR = 0.056, CFI = 0.930, TLI = 0.913, and χ²/df = 3.4, indicating a good fit to the data.

Conclusions: The Greek versions of the instruments demonstrated robust psychometric properties, confirming their reliability and validity for use in the local nursing context. Based on the observed deficits, particularly in domains related to data collection and evidence appraisal, the findings underscore the necessity of implementing structured training programs, integrating EBP content into undergraduate and postgraduate nursing curricula, and developing continuous professional development (CPD) initiatives focused on enhancing evidence-based decision-making competencies.

## Introduction

Evidence-based clinical practice (EBP) is a fundamental principle in improving the quality of health services, as it is directly linked to better outcomes for patients and more efficient use of available resources [1]. By integrating the latest research data into daily clinical practice, adverse events are reduced, clinical decision-making is improved, and patient safety is enhanced [1]. Furthermore, the implementation of EBP contributes to increasing the efficiency of services provided and reducing costs, creating a sustainable and high-quality health system [1]. Despite its proven benefits, its adoption remains a challenge due to barriers such as a lack of appropriate education and limited access to evidence-based sources [2]. In particular, health professionals are required to develop specialized skills in searching, evaluating, and integrating scientific knowledge into their daily practice [3]. The skills required include the critical evaluation of research data, understanding statistical methods, and the use of evidence-based care protocols. According to the literature, strengthening the capabilities of health professionals in EBP requires the formulation of appropriate educational programs and support policies at the organizational level [2,3]. The development of guided practice, participation in multidisciplinary teams, and systematic training can enhance the ability to implement EBP and reduce barriers to its adoption. Therefore, promoting educational initiatives and developing organizational policies that facilitate the implementation of EBP are essential to improving the health services provided [3].

EBP in nursing is a key pillar for improving the quality of care provided, contributing to both patient safety and the professional development of nurses [2]. The implementation of EBP allows for decision-making based on scientific evidence, which leads to better clinical outcomes, more efficient use of resources, and improved care processes [4]. Nurses who implement EBP can select the most appropriate technologies and therapeutic protocols, minimizing risks and enhancing the effectiveness of interventions [5]. Despite its benefits, the implementation of EBP in nursing practice faces significant challenges. One of the main obstacles is the difficulty of translating scientific knowledge into practical interventions, as there is often a lack of time and access to up-to-date literature [6]. Furthermore, resistance to change and adherence to traditional methods of care make it difficult to integrate new data into daily nursing practice [7]. To address these challenges, it is essential to continuously educate healthcare professionals, strengthen the research culture, and provide easy access to scientific resources. Promoting EBP in nursing is not only about improving patient care but also the overall progress of the profession. An environment that encourages the use of evidence for clinical decision-making leads to greater nurse confidence, more efficient management of available resources, and ultimately a more effective and humane healthcare system [8].

Creating an EBP-supportive environment through leadership engagement, interprofessional collaboration, and resource accessibility is essential for meaningful integration [9]. In the Greek context, EBP implementation remains fragmented. Although awareness of its importance has increased, empirical studies suggest moderate levels of knowledge and application among Greek nurses. For example, a national study among midwives revealed that only 43.5% were familiar with the term *evidence-based practice*, and most relied on colleagues rather than scientific databases for clinical guidance [10]. Similarly, among community nurses, knowledge and attitudes were moderately positive (mean 5.5/7), yet actual EBP implementation was limited (mean 4.5/7) [11].

One major obstacle in Greece has been the absence of validated instruments in the Greek language to assess EBP competencies and self-efficacy in nursing. Although some initial efforts have been made, such as the translation and adaptation of the Evidence-Based Practice Competency Questionnaire for Registered Nurses (EBP-COQ Prof©) [12] - their use has been limited, and they primarily assess broad domains such as EBP knowledge, attitudes, appraisal, and application in clinical decision-making [13].

This gap has restricted the ability of Greek healthcare institutions and educators to systematically evaluate EBP readiness, confidence, and integration. There is a pressing need for robust, psychometrically sound tools that can inform curriculum development, professional training programs, and organizational planning for EBP advancement. 

To address this gap in the Greek context, the present study aimed to translate, culturally adapt, and psychometrically validate two internationally recognized instruments: the Nursing Evidence-Based Practice Survey (NEBP Survey) and the Evidence-Based Practice Self-Efficacy Scale (EBP SES). The NEBP Survey focuses on organizational and contextual factors influencing EBP implementation in nursing settings, while the EBP-SES evaluates nurses’ confidence in performing EBP-related tasks such as literature searching, critical appraisal, and application of evidence in practice [14,15]. The inclusion of the EBP-SES in this study is grounded in Bandura’s Social Cognitive Theory, which identifies self-efficacy as a core determinant of human behavior and learning. In the clinical context, self-efficacy refers to a nurse’s belief in their ability to engage in EBP activities effectively. The study by Bandura [16] highlights four sources of self-efficacy: mastery experiences, vicarious experiences, verbal persuasion, and emotional states. These dimensions are reflected in the structure of the EBP-SES, which makes it an appropriate tool to assess psychological readiness for behavior change and skill adoption. Understanding self-efficacy in relation to EBP is crucial for designing targeted interventions, educational curricula, and leadership strategies aimed at fostering evidence-informed nursing practice.

By providing culturally adapted, theoretically grounded, and psychometrically sound tools in the Greek language, this study offers essential infrastructure for advancing evidence-based nursing education, research, and practice. Cross-cultural validation requires more than direct linguistic translation. Cultural adaptation ensures that items are conceptually and contextually appropriate for the target population, taking into account differences in educational systems, professional hierarchies, healthcare infrastructure, and prevailing attitudes toward research and evidence use. The validated instruments can support academic institutions, healthcare organizations, and policymakers in assessing EBP capacity, planning continuing professional development, and promoting a research-informed nursing workforce in Greece.

## Materials and methods

The sample size of 222 registered nurses was deemed adequate for psychometric evaluation and particularly appropriate for confirmatory factor analysis (CFA), which requires sufficient statistical power and model stability. Methodological guidelines recommend a participant-to-item ratio of at least 5:1 to 10:1 to ensure appropriate factor loading and model fit. Given that the NEBP Survey and the EBP Self-Efficacy Scale consist of 15 and 17 items, respectively, a minimum required sample size would range from 160 to 320 participants; thus, our sample meets the lower threshold [17,18]. Future studies could consider formal power analysis based on expected effect sizes to further validate sample adequacy. Inclusion criteria were as follows: (1) registered nurses actively working in clinical or community healthcare settings in Greece, (2) proficiency in the Greek language, and (3) voluntary participation with informed consent. Educators and nurse managers were excluded to ensure that responses reflected the experiences of frontline nursing staff most directly involved in evidence-based care delivery. Additional exclusion criteria included: (1) nurses not currently practicing (e.g., on leave or retired), (2) non-nursing professionals, and (3) incomplete questionnaire responses.

Instruments

A three-part questionnaire was used (see Appendix). The first section collected demographic and professional data, including age, gender, marital status, job title, years of clinical experience, and educational background (e.g., current or planned enrollment in a master's program). Participants were also asked whether they were aware of any evidence-based practice (EBP) initiatives at their workplace, to explore associations between personal/professional characteristics and EBP self-efficacy.

The NEBP Survey, developed by the University of Iowa Hospitals and Clinics and revised in 2005 and 2019, includes 15 items grouped into five subscales: Practice Climate, Data Collection, Evidence Appraisal, Implementation, and Access to Evidence. Each item is rated on a 5-point Likert scale, with higher scores indicating stronger perceptions of EBP readiness. The tool has demonstrated high internal consistency and structural validity in prior studies [14].

The EBP-SES, developed by Tucker et al. [13], consists of 17 items measuring confidence in executing core EBP activities: question formulation, literature search, critical appraisal, implementation, and evaluation. Responses are recorded on a scale from 0 (not at all confident) to 100 (completely confident), with higher average scores indicating greater EBP self-efficacy. The tool has shown excellent internal consistency in international validation studies (Cronbach’s α > 0.90).

Permission to translate and adapt both tools was obtained from the original developers.

The questionnaire translation process followed the steps suggested by Sousa and Rojjanasrirat [15], which include double independent translation, version reconciliation, back-translation, peer review, and pilot testing for cultural adaptation of the tool. This process ensures linguistic accuracy and the maintenance of the conceptual equivalence of the original questionnaire. To statistically assess the repeatability of the measurements between the test and retest, Pearson’s correlation coefficient and paired samples t-tests were conducted separately for each instrument. For the NEBP Survey, the correlation coefficient was *r* = 0.87, *P* < 0.001, and the paired t-test revealed no significant difference between administrations (*t*(19) = 0.68, *P* = 0.51). For the EBP Self-Efficacy Scale, the correlation coefficient was r = 0.92, p < 0.001, with the paired t-test again showing no significant difference (t(19) = 0.74, p = 0.47). These findings support the high test-retest reliability and temporal stability of the Greek versions of both instruments.

Data collection procedure

Data were collected through a structured online survey, which was created using a secure electronic platform. The survey included an introductory information sheet outlining the study's purpose, procedures, rights of participants, and informed consent statement. Participants were required to indicate consent before proceeding to the questionnaire. A purposive sampling strategy was used to recruit registered nurses working in clinical and community settings across Greece. The survey link was distributed via professional nursing associations, hospital mailing lists, and relevant social media groups. To prevent multiple submissions, each respondent was allowed to complete the survey only once per device. Data collection took place over four weeks in September and October 2024. Responses were automatically recorded in an encrypted database and were completely anonymous, ensuring participants’ confidentiality and adherence to ethical standards.

Data analysis was performed using SPSS statistical software (version 26.0; IBM Corp., Armonk, NY) for descriptive and inferential statistical analysis, while CFA was performed using JASP software (version 0.18.3). For the summary description of the sample, means, standard deviations, minimum and maximum values ​​for continuous variables, and absolute as well as relative frequencies for categorical variables were used. The internal consistency of the scales was assessed using Cronbach's α, where values ​​greater than 0.70 were considered satisfactory, while values ​​above 0.90 indicated excellent reliability. The structural suitability of the tools was assessed by CFA, taking into account the χ²/df, Goodness-of-Fit Index (GFI), Standardized Root Mean Square Residual (SRMR), Comparative Fit Index (CFI), and Tucker-Lewis Index (TLI). Good fit was considered when the GFI, CFI, and TLI values ​​were equal to or greater than 0.90, while the SRMR was less than or equal to 0.08 and the χ²/df ratio was less than or equal to 5, according to the criteria proposed in the literature [17]. For all statistical tests, the level of statistical significance was set at p < 0.05.

Ethical considerations

This study received approval from the Internal Ethics and Ethics Committee of the Department of Nursing of the University of Thessaly (No Prot.: 14/06-10-09-2024), at the meeting of October 8, 2024. The study was conducted following the principles of the Declaration of Helsinki (World Medical Association Declaration of Helsinki, 2013) on ethics in medical research involving human subjects. All participants were fully and thoroughly informed about the purpose, procedures, and their rights through written information, and voluntarily provided their written consent before their participation. Participation was completely voluntary, and participants retained the right to withdraw from the study at any stage, without any negative consequences or obligations. Data were collected anonymously, and the principles of personal data protection were fully respected, under the General Data Protection Regulation (GDPR, EU 2016/679).

## Results

The final sample included 222 registered nurses with diverse demographic and professional backgrounds. The mean age was 38.74 years (SD = 7.7). In terms of gender, 82 participants (37.1%) were male and 139 (62.9%) were female. Regarding marital status, the majority were married (117, 52.9%), followed by single (59, 26.7%), cohabiting (26, 11.8%), divorced (18, 8.1%), and widowed (1, 0.5%).

Participants reported a mean of 12.07 years (SD = 7.4) of professional experience. Regarding their current roles, 114 nurses (51.6%) identified as clinical nurses, while 107 (48.4%) were employed in school-based or community nursing positions. These categories reflect the major employment sectors represented in the sample; however, additional roles (e.g., research or administrative functions) may also be present but were not specifically captured.

Concerning educational aspirations, 100 participants (45.2%) expressed a desire to pursue a postgraduate degree, 2 (0.9%) were uncertain, and 119 (53.8%) did not intend to pursue further studies. At the time of the survey, 104 participants (47.1%) were already enrolled in a postgraduate program. Regarding institutional support, 120 nurses (54.3%) reported awareness of EBP programs within their organizations, while 101 (45.7%) reported no such awareness (Table 1).

**Table 1:** Demographic and professional characteristics of the sample.

Variable	Category	*n* (%)
Gender	Male	82 (37.1)
	Female	139 (62.9)
Marital status	Single	59 (26.7)
	Divorced	18 (8.1)
	Married	117 (52.9)
	Cohabitation	26 (11.8)
	Widowed	1 (0.5)
Working position	Clinical nurse	114 (51.6)
	School nurse	107 (48.4)
I intend to obtain a postgraduate degree	Yes	100 (45.2)
	Not sure	2 (0.9)
	No	119 (53.8)
I’m enrolled in a Master's course	Yes	104 (47.1)
	No	117 (52.5)
I’m aware of EBP programs implemented in my organization	Yes	120 (54.3)
	No	101 (45.7)

The descriptive statistics of the two instruments indicate generally favorable perceptions of EBP and moderate-to-high self-efficacy among Greek nurses. For the NEBP Survey, mean scores across subscales ranged from 3.68 to 4.13 on a 5-point Likert scale. The highest mean was observed in the Practice Climate subscale (M = 4.13, SD = 0.57), suggesting that many participants perceive their organizational environment as supportive of EBP. Conversely, the Data Collection subscale had the lowest mean (M = 3.68, SD = 0.94), indicating more variability in nurses' confidence or ease in systematically gathering clinical data area where additional training may be needed. Other subscales, including Implementation (M = 3.95, SD = 0.71) and Access to Evidence (M = 3.89, SD = 0.78), were rated moderately high, suggesting that while nurses report some ability to apply evidence and retrieve relevant resources, barriers may still exist. The total NEBP scale score was M = 3.88 (SD = 0.66), which is comparable to prior international studies using this tool, where values between 3.7-4.0 were associated with a moderate level of EBP integration. For the EBP SES, the mean score was 73.4 (SD = 14.0) on a 0-100 scale, indicating moderate to high levels of confidence in engaging in EBP-related tasks. This result aligns with similar validation studies conducted in other cultural contexts, where mean self-efficacy scores typically ranged between 65 and 80. Internal consistency reliability was high across all subscales. Cronbach’s α ranged from 0.78 to 0.96, with the total NEBP scale demonstrating excellent reliability (α = 0.95) and the EBP SES showing similarly strong internal consistency (α = 0.96). These values are within or above accepted thresholds for psychological and behavioral measures and are comparable to the original validation studies. These findings confirm that both tools demonstrate strong internal reliability and are appropriate for measuring EBP attitudes and self-efficacy in the Greek nursing population (Table 2).

**Table 2:** Descriptive statistics of the scales.

	Mean	Std. deviation	Minimum	Maximum	Cronbach's α
Practice Climate	4.13	0.57	2.40	5.00	0.86
Data Collection	3.68	0.94	1.00	5.00	0.94
Evidence Appraisal	3.78	0.81	1.00	5.00	0.92
Implementation	3.95	0.71	2.00	5.00	0.85
Access to Evidence	3.89	0.78	1.00	5.00	0.78
Nursing Evidence-Based Practice Total	3.88	0.66	1.68	5.00	0.95
Nursing Evidence-Based Practice Self-Efficacy	73.4	14.0	21.18	100.00	0.96

The CFA results indicated that both models demonstrated an acceptable to good fit, supporting the structural validity of the Greek versions. The five-factor model of the NEBP showed a χ²/df ratio of 4.1, which, while slightly above the ideal cutoff of ≤3, remains within the acceptable threshold for complex models [19]. The GFI was 0.960, and the SRMR was 0.041, both suggesting a good model fit. The CFI and TLI were 0.899 and 0.885, respectively, slightly below the recommended 0.90 threshold [20]. Despite these marginal deviations, the five-factor structure was retained due to its theoretical coherence, consistency with the original instrument, and strong performance in key indices. The model also preserves the interpretability and multidimensionality necessary for practical use in organizational assessment. In contrast, the single-factor model of the EBP SES demonstrated a better overall fit, with a χ²/df ratio of 3.4, GFI = 0.980, and SRMR = 0.056. The CFI and TLI exceeded the acceptable threshold, at 0.930 and 0.913, respectively, confirming a strong unidimensional structure consistent with the original scale. These results support the construct validity of both tools. The EBP SES exhibits excellent fit across all indices, while the NEBP, though slightly below optimal fit in some parameters, remains conceptually and psychometrically robust for use in both research and practice (Table 3).

**Table 3:** Goodness-of-fit indicators for the models of the scale used in the study.

	X^2^/df	Goodness-of-fit index (GFI)	Standardized Root Mean Square Residual (SRMR)	Comparative fit index (CFI)	Tucker-Lewis Index (TLI)
Nursing Evidence-Based Practice Five-Factor Model	4.1	0.960	0.041	0.899	0.885
Nursing Evidence-Based Practice Self-Efficacy Single-Factor Model	3.4	0.980	0.056	0.930	0.913

## Discussion

The findings of this study demonstrate that the Greek versions of the NEBP and EBP SES scales exhibit strong psychometric properties, confirming their reliability, structural validity, and practical applicability. This study is the first, to our knowledge, to perform a cultural adaptation and psychometric validation of both instruments simultaneously in the Greek nursing context. This dual validation fills a significant gap in the international literature, as prior Greek studies have largely focused on partial tools or self-constructed items to assess EBP-related competencies. 

The model fit analysis results for the Nursing Evidence-Based Practice Five-Factor Model (Χ²/df = 4.1, GFI = 0.960, SRMR = 0.041, CFI = 0.899, TLI = 0.885) and the NEBP Self-Efficacy Single-Factor Model (Χ²/df = 3.4, GFI = 0.980, SRMR = 0.056, CFI = 0.930, TLI = 0.913) indicate satisfactory to good fit of the models to the data. According to the recommended guidelines of the international literature, GFI and CFI values ​​above 0.90, SRMR below 0.08, and TLI close to or above 0.90 are considered indicators of good fit [18-20]. In particular, high GFI and very low SRMR confirm that the models represent the data satisfactorily. The SES model appears to achieve a better overall fit, with GFI = 0.980 and CFI = 0.930, which is consistent with the view that self-efficacy is a relatively coherent and unidimensional psychological factor [13]. In contrast, the five-factor model of EBP exhibits slightly lower CFI and TLI, reflecting the greater complexity and multidimensional nature of EBP in the clinical setting [3,6]. Overall, the results support the theoretical validity of the two instruments in nursing populations. The mean score of our sample on the NEBP SES was 73.4 (SD = 14.0), on a scale of 0-100, with excellent internal consistency (Cronbach's α = 0.96). These results are consistent with the findings of the initial development study of the tool by Tucker et al. [13], which reported a mean score of 73.5 (SD = 14.9) and Cronbach's α = 0.96, confirming the stability and reliability of the instrument. The high mean value of our sample reflects positive self-esteem of nurses in their ability to implement EBP, an element considered a critical predictive factor for the actual implementation of EBP in clinical practice [13].

High self-efficacy of nurses in implementing EBP is a critical factor in increasing the use of evidence-based interventions in daily clinical practice [21]. Similar findings have been reported in other studies, where high levels of self-confidence are associated with greater adoption of EBP and better clinical outcomes [22]. The high mean score observed in the EBP-SES (M = 73.4, SD = 14.0) reflects a generally positive perception of self-efficacy among Greek nurses regarding their ability to apply EBP in practice. As Bandura’s theory of self-efficacy posits, individuals who perceive themselves as capable are more likely to take initiative and persist in applying complex skills such as evidence appraisal and implementation in clinical settings [16]. However, self-efficacy alone is not sufficient; it must be nurtured through targeted, practice-oriented educational interventions and a supportive organizational culture [3]. In this context, findings from other disciplines reinforce the importance of structured, competency-based education in strengthening professional confidence and learning outcomes. For example, Ogut et al. [23] demonstrated that the integration of Special Study Modules (SSMs) in medical curricula significantly improved students’ academic engagement, self-efficacy, and perceived clinical competence in cross-sectional anatomy. This underscores a broader educational principle: that immersive, structured, and skill-based modules contribute to increased self-confidence and more effective clinical decision-making. Drawing parallels to nursing education, this finding supports the call for tailored, evidence-based training programs that not only enhance knowledge but also build enduring confidence in the practical application of EBP [23]. Moreover, these educational improvements are not merely theoretical. They are associated with concrete clinical outcomes, including improved diagnostic accuracy and more consistent application of evidence-based protocols in real-world settings. Therefore, beyond validating the EBP-SES, our study supports the integration of structured, modular learning opportunities-akin to SSMs-to enhance both the cognitive and affective domains of professional nursing development [23].

Overall, the data confirm that the EBP SES is a stable and reliable tool for measuring nursing self-confidence in the implementation of EBP in different clinical settings. The high mean value in *Practice Climate* (M = 4.13) and *Implementation* (M = 3.95) highlights a supportive organizational environment for the implementation of EBP, a finding that is consistent with international and Greek studies. Research has shown that the existence of a positive climate and organizational culture that promotes EBP is a key factor in its successful adoption [3,12]. In Greece, a study also recorded that nurses who perceive a positive climate in their unit report more frequent use of EBPs. However, the lower mean value in *Data Collection* (M = 3.68) is worrying and consistent with previous findings both abroad and in Greece, where the systematic collection and use of data for documentation remains a challenge due to a lack of training, time, and available resources [24]. Therefore, the findings confirm the international literature that, while the culture of support for EBP has improved, targeted interventions are required to strengthen data collection and analysis skills.

Implications for practice

The findings highlight the urgent need for context-specific educational initiatives aimed at improving nurses’ competencies in evidence-based data collection and critical appraisal. Beyond traditional workshops, integration of EBP content into undergraduate and postgraduate nursing curricula through simulation, case-based learning, and digital learning platforms may enhance both knowledge and applied skills. Organizational strategies should also be emphasized. These include the development of EBP champions within clinical units, access to point-of-care evidence databases, and protected time for evidence review and implementation. Additionally, structured mentoring programs involving senior nurses or interprofessional teams could further enhance individual self-efficacy and facilitate the practical application of EBP. To support sustainable change, hospitals and healthcare institutions may consider embedding EBP metrics into quality assurance systems, promoting an ongoing feedback culture where data use becomes part of routine practice. The adaptation of mobile applications that simplify literature searches, evidence rating, and clinical decision pathways could be a cost-effective tool to operationalize EBP [25,26].

Strengths of the study

This study possesses several notable strengths. First, it is among the very few efforts to simultaneously translate, culturally adapt, and psychometrically validate two internationally recognized instruments NEBP Survey and the EBP SES, within the same national nursing population. This dual validation provides a comprehensive framework for assessing both individual and organizational dimensions of EBP implementation. Second, the robust sample size (*n* = 222) ensures sufficient power for confirmatory factor analysis and enhances the generalizability of the psychometric findings. Third, the use of a validated, multistep translation protocol supports the conceptual and cultural equivalence of the instruments, ensuring their relevance for Greek nurses. Moreover, the study provides concurrent validity evidence through comparison with the original scales and recent international literature, strengthening its external consistency. Finally, the findings offer practical utility, as they equip researchers, educators, and healthcare administrators in Greece with valid tools to assess and improve EBP competencies at both the individual and institutional levels.

Limitations of the study

Several limitations should be acknowledged. First, the use of convenience sampling limits the generalizability of the findings, particularly across different healthcare sectors or regions in Greece. Second, the exclusive reliance on self-report measures introduces potential for social desirability bias, particularly given the professional nature of the topics assessed. Third, the cross-sectional design precludes causal inferences regarding the relationship between self-efficacy and actual EBP behavior. Additionally, although internal consistency was high, extremely elevated Cronbach’s α values (e.g., >0.95) may signal item redundancy or a lack of response variability. The modest test-retest sample (*n* = 20), while methodologically supported in scale development literature, could still be expanded in future research to further confirm temporal stability. Finally, while the translation process followed international guidelines, cultural nuances may still impact item interpretation, and future qualitative studies could explore how nurses conceptualize and enact EBP within specific clinical settings.

## Conclusions

The present study demonstrated that nurses display positive attitudes and high self-efficacy toward the implementation of EBP, with the work climate playing a decisive role. Despite the encouraging findings, areas that need further strengthening were identified, such as the collection and evaluation of evidence.

The development of educational and organizational strategies is necessary to ensure the full integration of EBP into daily clinical practice. Future research with larger and more representative samples will contribute to a deeper understanding of the factors that promote or hinder EBP.

## Appendices

Appendix

Φόρμα Συγκατάθεσης και Οδηγίες Συμπλήρωσης

Αγαπητέ/ή συνάδελφε,

Στο πλαίσιο μεταπτυχιακής μελέτης με αντικείμενο την εφαρμογή της τεκμηριωμένης νοσηλευτικής πρακτικής και την ετοιμότητα των επαγγελματιών υγείας για υιοθέτηση κλινικών παρεμβάσεων βασισμένων σε αποδεικτικά δεδομένα, σας προσκαλούμε να συμμετάσχετε ανώνυμα στην παρούσα έρευνα.

Η συμμετοχή σας είναι εθελοντική, δεν εμπεριέχει κανέναν απολύτως κίνδυνο ή επιβάρυνση και τα δεδομένα που θα καταγραφούν θα χρησιμοποιηθούν αποκλειστικά για ερευνητικούς σκοπούς, με απόλυτη εμπιστευτικότητα και ανωνυμία. Μπορείτε να αποχωρήσετε οποιαδήποτε στιγμή χωρίς καμία επίπτωση.

✅ Οδηγίες Συμπλήρωσης

Παρακαλούμε συμπληρώστε τα δημογραφικά στοιχεία που αφορούν το φύλο, την οικογενειακή σας κατάσταση, το εκπαιδευτικό επίπεδο και τη θέση εργασίας σας.

Στην κύρια ενότητα του ερωτηματολογίου:

Θα σας ζητηθεί να αξιολογήσετε τον βαθμό σιγουριάς (σε ποσοστό %) σχετικά με την ικανότητά σας να εκτελέσετε συγκεκριμένες νοσηλευτικές δραστηριότητες σχετικές με την τεκμηριωμένη πρακτική.

Ακολουθεί μια σειρά δηλώσεων, τις οποίες καλείστε να βαθμολογήσετε από 1 έως 5:

1 = Διαφωνώ απόλυτα

2 = Διαφωνώ

3 = Ούτε συμφωνώ ούτε διαφωνώ (Αβέβαιος/η)

4 = Συμφωνώ

5 = Συμφωνώ απόλυτα

Δεν υπάρχουν "σωστές" ή "λάθος" απαντήσεις. Μας ενδιαφέρει η πραγματική σας άποψη και εμπειρία.

Η συμπλήρωση του ερωτηματολογίου δεν θα διαρκέσει περισσότερο από 10-15 λεπτά.

Με την ολοκλήρωση και αποστολή του ερωτηματολογίου, τεκμαίρεται ότι έχετε ενημερωθεί επαρκώς και συναινείτε στη συμμετοχή σας στην έρευνα.

Σας ευχαριστούμε θερμά για τον χρόνο και τη συμβολή σας στη βελτίωση της νοσηλευτικής φροντίδας βασισμένης σε τεκμηριωμένα δεδομένα.

**Table 4:** Δημογραφικά στοιχεία

Δημογραφικά στοιχεία		
Φύλο	Άντρας	
	Γυναίκα	
Οικογενειακή κατάσταση	Άγαμος/η	
	Έγγαμος/η	
	Διαζευγμένος/η	
	Σε συμβίωση	
	Χήρος/α	
Εκπαιδευτικό επίπεδο	Πτυχίο νοσηλευτικής ΤΕ/ΠΕ	
	Μεταπτυχιακό / Διδακτορικό	
Τρέχουσα θέση εργασίας	Κλινικός νοσηλευτής/τρια	
	Σχολικός νοσηλευτής/τρια	

**Table 5:** Greek translation of the Evidence-Based Practice Self-Efficacy Scale (EBP-SES). Permission was obtained to translate the EBP-SES into Greek, provided that the items and response format remained unchanged [13].

	0 10 20 30 40 50 60 70 80 90 100 καθολου σίγουρη μέτρια σίγουρος/η Σίγουρος/η
	Είμαι ΤΟΣΟ (%)ΕΠΙ ΤΗΣ ΕΚΑΤΟ σίγουρος/η ότι μπορώ να ολοκληρώσω τις παρακάτω δραστηριότητες σχετικές με την νοσηλευτική πρακτική:
1	Κάνω συχνά ερωτήσεις σχετικά με την πρακτική μου %
2	Εντοπίζω τους διαθέσιμους πόρους στο τμήμα μου και γενικότερα το ίδρυμά που εργάζομαι ώστε να διευκολύνω την κατανόηση της ερευνητικής βιβλιογραφίας που σχετιίζεται με τη νοσηλευτική μου πρακτική. %
3	Εντοπίζω τους απαραίτητους πόρους στο τμήμα μου και στο ίδρυμά που εργάζομαι για να υλοποιήσω μια αλλαγή σε μια τεκμηριωμένη πρακτική %
4	Εντοπίζω και εξετάζω δημοσιευμένες κατευθυντήριες οδηγίες ορθής πρακτικής που αφορούν νοσηλευτικές παρεμβάσεις σημαντικές για την πρακτική μου %
5	Εντοπίζω και εξετάζω δημοσιευμένες ερευνητικές μελέτες που έχουν σχέση με νοσηλευτικές παρεμβάσεις σημαντικές για την πρακτική μου.. %
6	3Οργανώνω την απαραίτητη υποστήριξη και τις διαδικασίες για την πραγματοποίηση μιας αλλαγής στη νοσηλευτική πρακτική με βάση τα αποδεικτικά στοιχεία (έρευνα, κατευθυντήριες γραμμές κλινικής πρακτικής, κλινική εμπειρογνωμοσύνη, στόχοι/προτιμήσεις των ασθενών). %
7	Προσδιορίζω τακτικά τα αναμενόμενα αποτελέσματα των ασθενών για να θέσω στόχους στις νοσηλευτικές παρεμβάσεις. %
8	Ενσωματώνω διάφορες πηγές τεκμηρίωσης και τις εφαρμόζω στον πληθυσμό της ειδικότητάς μου και στην πρακτική μου. . %
9	Ενεργοποιώ τις διαδικασίες για την εφαρμογή μιας αλλαγής για τεκμηριωμένη πρακτική. . %
10	Τροποποιώ τις νοσηλευτικές παρεμβάσεις που συνιστώνται για τον πληθυσμό των ασθενών μου με βάση τα χαρακτηριστικά της συγκεκριμένης μονάδας στην οποία εργάζομαι. . %
11	Αξιολογώ τακτικά την ερευνητική βιβλιογραφία και άλλες πηγές τεκμηρίωσης σχετικά με τις νοσηλευτικές παρεμβάσεις για τον πληθυσμό της ειδικότητάς μου και την πρακτική μου.. . %
12	Εφαρμόζω συστηματικά νοσηλευτικές παρεμβάσεις που υποστηρίζονται από τεκμηρίωση (έρευνα και άλλες πηγές, όπως κατευθυντήριες οδηγίες) για τον πληθυσμό των ασθενών μου και την πρακτική μου. %
13	Τροποποιώ τις νοσηλευτικές παρεμβάσεις που εφαρμόζω κατά κανόνα, με βάση όσα μαθαίνω για τις προτιμήσεις του ασθενούς μου. . %
14	Τροποποιώ τακτικά τις νοσηλευτικές παρεμβάσεις με βάση τα αποτελέσματα και τους στόχους.. . %
15	Αξιολογώ τακτικά την αποτελεσματικότητα των νοσηλευτικών παρεμβάσεων χρησιμοποιώντας μετρήσιμα αποτελέσματα.. . %
16	Λαμβάνω την κατάλληλη κατάρτιση και εκπαίδευση ώστε να είμαι σε θέση να εφαρμόσω αποτελεσματικά μια τεκμηριωμένη νοσηλευτική παρέμβαση ή πρακτική.
17	Εφαρμόζω μια τεκμηριωμένη νοσηλευτική παρέμβαση, εξατομικευμένη στην κατάσταση του ασθενούς/οικογένειάς του, χωρίς να χάνω την αξιοπιστία της παρέμβασης (δηλ. την εφαρμόζω όπως προοριζόταν να εφαρμοστεί).. . %

**Table 6:** Greek translation of Nursing Evidence-Based Practice (NEBP) Survey. Permission was obtained to translate the NEBP survey into Greek, provided that the items and response format remained unchanged [14].

		Διαφωνώ απόλυτα	Διαφωνώ	Αβέβαιος	Συμφωνώ	Συμφωνώ απόλυτα
1.	Θεωρώ σημαντική την τεκμηριωμένη νοσηλευτική πρακτική	1	2	3	4	5
2.	Έχω εύκολη πρόσβαση σε περιοδικά νοσηλευτικής έρευνας.	1	2	3	4	5
3.	Γνωρίζω πού μπορώ να βρω τεκμηριωμένα στοιχεία (π.χ. ερευνητικά ευρήματα ή τεκμηριωμένες κλινικές κατευθυντήριες οδηγίες) για να καθοδηγήσω την πρακτική μου.	1	2	3	4	5
4.	Θα ήταν χρήσιμη η ύπαρξη μιας ομάδας μελέτης βιβλιογραφίας, με συζήτηση των ευρημάτων νοσηλευτικής έρευνας,.	1	2	3	4	5
5.	Η βοήθεια από κάποιον, στην αναζήτηση βιβλιογραφίας και στην απόκτηση άρθρων, θα αύξανε τη χρήση τεκμηριωμένων πρακτικών.	1	2	3	4	5
6.	Μπορώ να διαβάσω μια ερευνητική νοσηλευτική αναφορά και να έχω μια γενική άποψη για τα πλεονεκτήματα και τις αδυναμίες της..	1	2	3	4	5
7.	Μπορώ να διαβάσω μια έκθεση νοσηλευτικής έρευνας και να κρίνω σωστά την επιστημονική της αξία.	1	2	3	4	5
8.	Μπορώ να ασκήσω κριτική σε αναφορές «σύνθεσης» ή τεχνολογικές αξιολογήσεις (π.χ. συστηματικές ανασκοπήσεις) για μια γενική κατανόηση των πλεονεκτημάτων και των αδυναμιών τους..	1	2	3	4	5
9.	Στον χώρο που εργάζομαι, θα ήταν χρήσιμος ένας πίνακας ανακοινώσεων για την ανταλλαγή ερευνητικών άρθρων.	1	2	3	4	5
10.	Είμαι πρόθυμος/η να δοκιμάσω νέες καινοτομίες που αποδεικνύονται αποτελεσματικές.	1	2	3	4	5
11.	Κατανοώ τη διαδικασία εφαρμογής τεκμηριωμένης πρακτικής στον χώρο που εργάζομαι.	1	2	3	4	5
12.	Γνωρίζω αποτελεσματικές στρατηγικές για την εφαρμογή αλλαγών στην πρακτική.	1	2	3	4	5
13.	Συμμετέχω στη συλλογή δεδομένων για ερευνητικές μελέτες (π.χ. διεξαγωγή ερευνών, όχι προγράμματα για την εφαρμογή τεκμηριωμένης πρακτικής).	1	2	3	4	5
14.	Συμμετέχω στη συλλογή δεδομένων για προγράμματα βελτίωσης ποιότητας.	1	2	3	4	5
15.	Συμμετέχω στη συλλογή δεδομένων για προγράμματα τεκμηριωμένης πρακτικής.	1	2	3	4	5
